# Omega-3 Fatty Acids in Pregnancy—The Case for a Target Omega-3 Index

**DOI:** 10.3390/nu12040898

**Published:** 2020-03-26

**Authors:** Clemens von Schacky

**Affiliations:** 1 Preventive Cardiology, Ludwig Maximilians-University Munich, Ziemssenstr. 1, 80336 München, Germany; clemens.vonschacky@med.uni-muenchen.de; Tel.: +49-89-44005-2165; Fax: +49-89-44005-2192; 2 Omegametrix, Martinsried, Am Klopferspitz 19, 82152 Planegg, Germany

**Keywords:** eicosapentaenoic acid, docosahexaenoic acid, pregnancy, lactation, premature birth

## Abstract

Scientific societies recommend increasing intake of docosahexaenoic acid (DHA) by 200 mg/day during pregnancy. However, individually, clinical events correlate quite strongly with levels of eicosapentaenoic acid (EPA) and DHA in blood, but these levels poorly correlate with amounts ingested. EPA and DHA in erythrocytes (Omega-3 Index) have a low biologic variability. If analyzed with a standardized analytical procedure (HS-Omega-3 Index^®^), analytical variability is low. Thus, the largest database of any fatty acid analytical method was provided. Pregnant women in Germany had a mean Omega-3 Index below the target range suggested for cardiovascular disease of 8–11%, with large interindividual variation, and quite independent of supplementation with EPA and DHA. In Germany, premature birth is a major health issue. Premature birth and other health issues of pregnant women and their offspring correlate with levels of EPA and DHA in blood and can be reduced by increasing intake of EPA and DHA, according to individual trials and pertinent meta-analyses. Very high intake or levels of EPA and DHA may also produce health issues, like bleeding, prolonged gestation, or even premature birth. While direct evidence remains to be generated, evidence from various scientific approaches supports that the target range for the Omega-3 Index of 8–11% might also pertain to pregnancy and lactation.

## 1. Introduction

In many countries, pregnant women or women of child-bearing age rarely consume foods suitable as sources for the long-chain Omega-3 fatty acids eicosapentaenoic acid (EPA) or docosahexaenoic acid (DHA) [1,2,3,4]. In pregnancy, vegetarian and vegan women continue to avoid fish, the typical source of EPA and DHA, which puts them at risk for maternal undernutrition and impaired fetal growth [5]. Vegetarians and vegans have a low Omega-3 Index [6]. Thus, vegetarian and vegan pregnant women have low plasma levels of EPA and DHA [5]. Against this background, many authoritative bodies and expert scientific organizations recommended that pregnant women consume an extra 200 mg/day DHA, for example, as fatty fish from the sea once a week [7,8,9]. Consumption of long-living predatory fish, containing pollutants like methylmercury and/or organic toxins, was discouraged [8,9]. Intake of plant-derived alpha-linolenic acid was not recommended, since humans poorly convert this Omega-3 fatty acid to EPA, and are unable to convert EPA to DHA in sufficient quantities [7,8]. A reduction of premature birth before week 34, inconsistent results with respect to cognitive development of the child, and data suggesting a reduction of atopic diseases are usually cited in support of the recommendations [7,8,9,10].

## 2. Intake vs. Blood Levels

Clinical effects correlate less well with intake than with blood levels. An example is premature birth before week 34 of pregnancy, which is more likely with low levels than with high levels of EPA and DHA, with risk for preterm birth correlating inversely with levels in erythrocytes, whole blood, or plasma [11,12,13,14]. Data derived from assessments of intake of EPA and DHA showed smaller risks, if at all detected, and no association with dose [15]. Increasing consumption of EPA and DHA in an untargeted manner (i.e., not guided by level assessments) reduced premature birth before week 34 of pregnancy by 42%, according to a recent Cochrane meta-analysis [16]. Taken together, current data indicate that low levels of EPA and DHA might cause premature birth, providing an argument to determine them before or in pregnancy [11,12,13,14,15,16].

That clinical effects correlate less with intake than with blood levels is partly explained by issues of bioavailability of EPA and DHA [17,18,19]:

- high bioavailability with a high-fat meal compared to a low-fat meal (up to factor 13)
- a large interindividual variability of uptake of ingested EPA and DHA (up to factor 13)
- matrix effects, that is, dependence on other compounds ingested with EPA and DHA (up to factor 10)
- other issues of bioavailability, like an attenuated response to supplementation with EPA and DHA in women with obesity prepregnancy, compared to lean women [20].

Taken together, intake is only a weak predictor of levels in an individuum (e.g., [21]). Therefore, it is very difficult, if not impossible, to predict the increase of levels in response to a given dose in an individual. In keeping with this, using a uniform dose to achieve a clinical effect in all pregnant women, like prevention of premature birth, cannot be an efficient measure. Using arithmetic, it is possible to calculate a dose response in a large population [22]. However, individual women, and not populations, get pregnant.

Blood levels of EPA and DHA can be assessed in many fatty acid compartments: whole blood, plasma, plasma phospholipids, platelets, erythrocytes, and many others. The percentage of EPA and DHA of a total of 26 fatty acids measured in erythrocytes (Omega-3 Index) is a long-term parameter, has a low biologic variability, and correlates with EPA and DHA in tissue [17,23]. For fatty acids in erythrocytes, there is a standardized analytical procedure („HS-Omega-3 Index^®^“), supported by some 300 publications, the largest database of all fatty acid analytical procedures [17,23]. EPA and DHA plasma and plasma phospholipids rather reflect short-term changes of fatty acids, and therefore have a high biologic variability [17,23]. As yet, no standardized analytical procedure has been suggested for any other fatty acid compartment.

Of note, neither in pregnant women nor in any other individual has an Omega-3 Index <2% ever been found in erythrocytes (as different from dried blood spots), indicating that human life without EPA and DHA is not possible [24]. This puts EPA and DHA in the same category as water or salt and questions the wisdom of uncritically using scientific tools devised for drugs to investigate the effects of EPA and DHA. Among these tools are placebo-controlled intervention trials using a uniform dose without determining baseline and on trial levels of EPA and DHA, and their pertinent meta-analyses. This matter is discussed in more detail elsewhere [17,25].

Moreover, in pregnant women, as in all human beings, and in keeping with the baseline and bioavailability issues just mentioned, levels of EPA and DHA vary substantially from person to person: in pregnant women, individual levels of EPA and DHA ranged from 3.81% to 11.10% (details in Table 1) [26].

Taken together, the exact impact of intake of EPA and DHA on the Omega-3 Index is unpredictable in an individuum, but clinical effects depend on the Omega-3 Index. Therefore, to define the necessary intake of EPA and DHA, not a dose but a target Omega-3 Index is decisive.

## 3. Target Omega-3 Index in Pregnancy

The placenta actively transports fatty acids, usually from the pregnant woman to the fetus [27]. This transport aims to achieve 8–9% DHA in total fatty acids in the membranes of the erythrocytes of the fetus [27,28,29,30]. Without substitution, a pregnant woman’s DHA levels will fall in proportion to the amounts transported to the fetus, despite mobilization of DHA with advancing gestation [28,29,31]. Longer pregnancies result in higher DHA levels in erythrocytes in the child [32,33]. After birth, mother’s milk can be enriched with EPA and DHA through diet [34]. With an Omega-3 Index of 8%, the lactating mother will produce milk containing 1% EPA and DHA [35]. The data indicate that the placenta aims for an Omega-3 Index for the fetus higher than the Omega-3 Index found in most pregnant women, at least in Germany [26,27,28,29,30]. Less data are available for lactation. In order to maintain an optimal Omega-3 Index, and not to impoverish DHA, an expectant mother’s Omega-3 Index should be maintained at around 10%.

Currently, some 7% of all births in Germany are before week 37 of gestation, and some 2% before week 34 of gestation [36]. Thus, premature birth is a major health issue in Germany. In a semi-representative assessment of pregnant and lactating women in Germany, using the HS-Omega-3 Index method, the mean Omega-3 Index of all women assessed was 6.23 ± 1.48% (Table 1) [26]. Pregnant women had higher levels than lactating women, indicating that the active transport of DHA by placenta to the fetus left the lactating mother depleted (Table 1). The placenta’s active transport of DHA does not lower a mother’s DHA levels below some 8%, in case they are maintained sufficiently high [28,29]. Thus, on average, the current state of pregnant women in Germany in terms of EPA and DHA is insufficient, probably both for mother and child. This is supported by the rate of premature birth in Germany just mentioned [36]. The data also indicate that a DHA level of 5% in erythrocytes, as previously suggested for pregnant women, is inadequate [15], and that the target for DHA is between 8% and 9%, with EPA probably adding another percent or two, that is, the data suggest a target range for the Omega-3 Index in pregnancy of 8–11%, as for all other health issues so far investigated (with higher levels probably needed for chronic inflammatory diseases).

Currently, in Germany, only some 11.5% of pregnant women supplement with Omega-3 fatty acids in pregnancy [26]. Similar data have been found in Israel [37]. Vegetarian and vegan pregnant women have been observed to have low plasma levels of EPA and DHA [5]. Diagnosing the need to supplement by determining the Omega-3 Index might serve to increase the number of pregnant women supplementing, since one reason for not supplementing is overestimating one’s own Omega-3 fatty status [38]. Diagnosing the need to supplement would conform the practical conclusion of the most recent Cochrane meta-analysis on Omega-3 fatty acids in pregnancy: “A universal strategy of supplementation may be reasonable, although, ideally, with more knowledge, this would be targeted to women who would benefit the most.” [16].

## 4. Limitations of Previous Intervention Trials

No human being, pregnant women included, has an Omega-3 Index <2% [24]. Therefore, other than drug trials, randomized intervention trials with Omega-3 fatty acid never test the presence of Omega-3s vs. their absence. As mentioned, clinical effects correlate with erythrocytes levels rather than with intake. Therefore, for a clinical effect to become detectable, not only should mean Omega-3 erythrocyte levels be higher in the verum group than in the placebo group, levels should also not overlap (i.e., individual levels in verum should be higher than individual levels in placebo or control). This would mimic the situation in a drug trial, or the situation that randomized controlled trials are designed for. Achieving this goal is seriously impeded by the issues in bioavailability discussed above. Moreover, baseline levels of participants should not be optimal, since in individuals with optimal baseline levels in erythrocytes, no effect of an intervention with Omega-3s can be expected. Usually baseline erythrocyte levels were not among the inclusion or exclusion criteria of randomized trials. Also, during a trial, these levels were usually not controlled. Overlapping of levels was thus not detected, and the dose of Omega-3s could not be corrected (e.g., increased) to achieve a separation of levels [17,25]. Also, problems of bioavailability were ignored. Taken together, the design of most previous intervention trials was biased towards not detecting an effect of Omega-3s. Since meta-analyses only summarize results of previous trials, many meta-analyses reported neutral results. Standardized measurements of EPA and DHA will make trials more resistant to the issues just discussed, as recently reported [36]. Thus, results of previous trials must be seen critically.

## 5. Clinical Benefit of Omega-3s in Pregnancy

As mentioned, supplementing Omega-3 fatty acid in pregnancy is being recommended by an impressive number of scientific societies, but not by all [7,8,9,39]. Usually, the evidence supporting the recommendation is reviewed to some extent in the recommendations [7,8,9,39]. Therefore, here, this evidence is briefly presented from the perspective of levels, rather than from the perspective of supplementation.

*Perinatal mortality, premature birth, complications for the mother*: In DOMINO, with 2399 participating pregnant women, one of the largest randomized intervention trials, the effect of 800 mg DHA and 100 mg EPA/day was compared to an Omega-3 fatty acid-free placebo [40]. This increased EPA and DHA in cord blood phospholipids from a mean of 6.4% to 8.0%, corresponding to a somewhat higher Omega-3 Index in erythrocytes [41]. Intake of DHA explained only 21% of the variability of levels measured in the verum group, again highlighting the loose relation between intake and levels. This also explains why some 80% of levels measured in verum and placebo groups overlapped (i.e., were found to be in the same range) [41]. Clinical effects, however, were substantial: In comparison to placebo, verum lowered premature birth before week 34 by 51% (RR 0.49, 95% CI 0.25 to 0.94), resulting in a higher mean birth weight by 68 g (95% CI, 23 to 114 g), fewer children had low birth weight (RR 0.65, 95% CI 0.44 bis 0.96), and fewer newborns needed intensive care [40]. Importantly, in the verum group, three newborns died, while 12 died in the placebo group [40]. Considering these data, it was probably less important that the primary endpoint of the trial, improvement of postpartum depression, was not met [40]. Some, but not all, parameters of cognitive function measured were improved [40]. Bleeding episodes were not more frequent in verum [40]. Data of the most recent pertinent Cochrane meta-analysis of a total of 70 randomized intervention trials with a total of 19,927 participants demonstrated similar but less pronounced results, with a reduction of premature birth before week 34 by 42%, in keeping with the discussion above (RR 0.58, 95% CI 0.44 to 0.77) [16]. Less children had low birth weight (14.0% instead of 15.6%; RR 0.90, 95% CI 0.82 to 0.99), and possibly less newborns needed medical attention (RR 0.92, 95% CI 0.83 to 1.03). Perinatal mortality was not significantly reduced (RR 0.75, 95% CI 0.54 to 1.03). Pre-eclampsia might have been reduced (RR 0.84, 95% CI 0.69 to 1.01), corresponding to epidemiologic data [42,43,44]. An improvement of postnatal depression was not found, nor were parameters of cognition improved in the child. Pregnancies were not significantly longer, nor were higher rates of induced labor or abdominal delivery found [16]. In the majority of trials, the doses of EPA and DHA used were considerably lower than 1 g/day, with the consequence that their levels probably overlapped in verum and placebo groups by more than 80% (see above), making it difficult to discern effects of the intervention. This probably explains why smaller effects were found in the Cochrane meta-analysis than in DOMINO. The results of the Cochrane meta-analysis therefore reflect the methodologic issues discussed above, indicating that positive effects of EPA and DHA on the parameters mentioned might be larger, and untoward effects smaller, in cases that use of EPA and DHA were to be guided by a properly defined target range. This is supported by the results of an older meta-analysis, which found a reduction in premature birth before week 34 by 58% (RR 0.42, 95%CI 0.27 to 0.66), a reduction of perinatal death by 49% (RR 0.51, 95%CI 0.26 to 1.01), an increase of duration of pregnancy by +1.95 weeks, and an increase of birth weight by 122.1 g (all significant) [45].

A meta-analysis of seven intervention trials on gestational diabetes demonstrated that EPA and DHA reduced fasting blood sugar (RR 0.56, 95%CI 0.87 to 0.24) and HOMA-IR (RR 0.52, 95%CI −0.83 to −0.21) statistically significantly in comparison to placebo, while significance was narrowly missed in the cases of macrosomia (RR 0.48, 95% CI 0.22 to 1.02) and hyperbilirubinemia (RR 0.46, 95%CI 0.19 to 1.10) [46].

Epidemiologic data show that the lower the levels of EPA and DHA in pregnant women, the more likely postpartum depression [47,48,49]. As mentioned above, the placenta transports EPA and DHA to the fetus, in many cases causing a deficit of EPA and DHA in the mother, the latter considered a risk factor for postpartum depression [50]. Such a deficit can pave the way for neuroinflammation and aberrant neurotransmission, also considered risk factors for postpartum depression [50]. While a recent meta-analysis of pertinent intervention trials found no effect of EPA and DHA during pregnancy on postpartum depression, a systematic review saw positive effects [11,50].

*Asthma, allergies, and aspects of cognition in the child*: In a quite large intervention trial, 2 g EPA and DHA per day given to pregnant women reduced asthma and persistent wheezing in their children by half (RR 0.47, 95% CI 0.26 to 0.48) [51]. It was not surprising that the effect was only detectable in children of mothers with low baseline levels. In children of mothers with high baseline levels, no effect was detectable; however, asthma and persistent wheezing in the placebo group corresponded to the therapeutic effect of EPA and DHA in the mothers with low baseline levels [51]. In a recent pertinent meta-analysis of seven intervention trials with 2047 children, it was confirmed in a slightly less pronounced manner that EPA and DHA in pregnancy reduce asthma and persistent wheezing in the child [46]. Epidemiologic studies and some, but not all, intervention trials demonstrated that EPA and DHA in pregnancy can reduce the propensity towards allergies in the children [52,53,54,55].

The children of the participants in the quite large intervention trial mentioned above had a follow-up examination at age 6, and a higher fat-free body mass and a higher bone mass was found in the verum group [56]. Meta-analyses on this topic complained about the quality of the trials and found a higher birth weight and waist circumference in newborns, but no effects in older children [57,58].

Prenatal supplementation with DHA improved parameters of heart rate variability, with effects depending on erythrocyte DHA [59,60], and appeared to mitigate the association between childhood overweight condition or obesity and blood pressure but did not reduce obesity itself [58,61]. Higher Omega-3 fatty acids in breast milk were associated with a lower blood pressure in the child [62].

It has been suggested that low levels of DHA at birth or in childhood may act as a modifiable risk factor for autism spectrum disorder and attention deficit hyperkinetic disorder [59,63,64]. Intervention trials have been conducted in children with autism spectrum disorder and were positive for social interaction, communication, and repetitive and restrictive interests and behaviors, but not for all symptoms [60,64,65]. Improvements have also been seen in children with attention deficit hyperkinetic disorder who were treated with EPA and DHA [61,66].

Higher EPA and DHA status at birth was associated with better childhood neurodevelopment, and meta-analyses of intervention trials with prenatal omega-3 fatty acids found parameters of cognition in children aged 2–5 improved, but not in other age groups [16,67]. In another meta-analysis of pertinent intervention trials, positive effects were seen on psychomotor and visual development of the child [68]. In a more recent trial, increases in maternal blood DHA concentration in pregnancy were related to higher IQs, but this effect disappeared when socio-economic status was statistically controlled [69].

## 6. Safety and Tolerability

According to the European Food Safety Authority, up to 5 g/day EPA and DHA are safe, which is also true for pregnant and lactating women [70]. In intervention trials in pregnant women, up to 2.7 g/day EPA and DHA were safe and tolerable [7,8,9,70]. A bleeding tendency caused by EPA and DHA was not seen in the Cochrane meta-analysis mentioned [16]. In an analysis of postoperative bleeding, higher levels of EPA and DHA were associated with fewer bleeding episodes [71]; after a thromboembolic event, patients on anticoagulation lived longer, but had no more bleeding episodes than patients with a lower Omega-3 Index [72]. However, in two cardiovascular trials with high doses of EPA, bleeding episodes occurred 0.1% more frequently per year in verum than in placebo [73,74]. From a pragmatic point of view, pregnant women should probably avoid daily doses of EPA and DHA >2.7 g/day [9,16,51], and/or an Omega-3 Index ≥16% to avoid bleeding.

Clearly, by reducing premature birth, a higher Omega-3 Index or higher intake of EPA and DHA will result in longer gestations, which has been reported in pertinent meta-analyses to be 1.42 days (95% CI 0.73 to 2.11) [16], and in an individual trial up to some 6 days [9,75,76]. Rates of medical induction of labor and of abdominal delivery were increased in one trial (RR 1.28, 95% CI 0.6 to 1.54, *p* = 0.01) [40,41]. However, post-term induction of labor or caesarean sections were not found to be more frequent in a recent large pertinent meta-analysis, but in one trial some participants reached very high levels [16,40,41]. In contrast, it has recently been reported that some premature births were associated with very high levels of EPA and DHA [13]. Of note, a Mendelian randomization study found little importance for DHA in determining length of gestation, while arachidonic acid seemed to have more relevance [50]. The ORIP trial specifically asked the question whether induced labor or caesarean sections would become more frequent by intervention with 900 mg omega-3 fatty acids beginning before week 20 up to week 34 of gestation, but saw no difference between the omega-3 and the control group [77]. Taken together, the risk of increasing induced labor or caesarean sections or premature birth by increasing intake of EPA and DHA might depend on a reduction of arachidonic acid, again supporting standardized measurements of erythrocyte fatty acids, also providing results for arachidonic acid.

Upon supplementation with DHA, levels of EPA rise [78,79]. EPA is among the fatty acids transported from mother to fetus by placenta [27]. Levels of EPA and DHA in plasma predicted premature birth, an effect probably partly mediated by EPA-derived eicosanoids [11]. Supplementation with EPA-rich oils during pregnancy or postpartum improved maternal depression, which DHA did not [16,80]. Thus, EPA and DHA are biologically not easily separated, and EPA appears to have some positive effects on its own in pregnancy, suggesting that a metric providing the levels of both EPA and DHA in pregnancy (i.e., the Omega-3 Index) has advantages over measuring only DHA [15].

It is a well-known phenomenon that, in erythrocytes, increasing levels of EPA and DHA (e.g., by means of diet) will decrease levels of arachidonic acid. Both erythrocyte arachidonic acid and DHA deficiencies were found to be associated with higher risk of neonatal morbidities and mortality in preterm infants [81]. Moreover, a post hoc analysis of blood levels in ORIP found some premature births to be associated with very high blood levels of EPA and DHA [13]. Therefore, an upper limit for the Omega-3 Index in pregnancy should be defined, and maybe also a lower limit for arachidonic acid. Based on the target range suggested for cardiovascular prevention, an upper limit of 11% seems prudent in order to not reduce arachidonic acid substantially [17,24]. Although large parts of the Japanese and South Korean populations have an Omega-3 Index in the target range of 8–11%, specific data need to be generated to support this upper limit and probably also to define a target for arachidonic acid [82,83].

As widely recommended, long-living predatory fish should not be used as a source of EPA and DHA because of their content of toxins [7,8,9]. Other sources are fish like salmon, mackerel, sardines, or supplements with oils from fish, krill, or algae. Taking Omega-3 supplements with the main meal maximizes bioavailability of EPA and DHA and minimizes typical adverse events like fishy hiccups.

## 7. Discussion

In no trial mentioned above did recruitment depend on baseline levels, and target levels were not defined. Therefore, it must be assumed that in a substantial proportion of the trials discussed above, blood levels during the trials overlapped to some degree, as demonstrated for the DOMINO trial [40]. Without proper measuring of levels during and/or at the end of the trial, it is impossible to identify the trials in which levels did or did not overlap. However, since an overlap of levels reduces effect size, it can be speculated that many trials underestimated the effects of omega-3 fatty acids, in cases where they found an effect. It can only be speculated that this also led to neutral results of other trials, although, again, this can only be clarified by properly measuring blood levels. Clearly, the issues just discussed combine to diminish effects seen in pertinent meta-analyses.

Detecting nutritional deficits before or early in pregnancy has repeatedly been recommended [7,8,9]. The Omega-3 Index reliably detects deficits in EPA and DHA, in comparison with other methods, more reliably so with the standardized analytical method HS-Omega-3 Index [17,23]. When deficits are detected, like in most pregnant women in Germany [26], a targeted increase of intake of up to 2.7 g/day controlled by measuring the standardized Omega-3 Index is more reliable and safer than an untargeted increase of intake with the potential of achieving an Omega-3 Index ≥16% (rare bleeding episodes) [73,74]. Safety and tolerability of EPA and DHA make the target range of 8–11% of the standardized Omega-3 Index safer than lower values, since this minimizes perinatal mortality, premature birth, and other complications of pregnancy, as asthma or persistent wheezing is minimized in the child. Moreover, mother and child may very well benefit in other ways, like aspects of brain function.

## 8. Conclusions

In many countries, intake of EPA and DHA by pregnant women or by women with child-bearing potential is low, which results in low levels of EPA and DHA (e.g., assessed in erythrocytes (Omega-3 Index)). This results in premature birth and many other health issues for mother and child. While it is recommended that pregnant women increase intake of DHA by 200 mg/day, the correlation between intake and resulting levels is poor, and many pregnant women have a low Omega-3 Index despite following the recommendation. Based on current evidence discussed above, I suggest that the target range for the Omega-3 Index of 8–11% might pertain to pregnancy and lactation. My suggestion needs to be substantiated by direct measurements.

## Figures and Tables

**Table 1:** Levels of eicosapentaenoic acid (EPA), docosahexaenoic acid (DHA), and the Omega-3 Index in pregnant and lactating women, with and without supplementation with EPA and DHA, in % of a total of 26 fatty acid analyzed using the HS-Omega-3 Index method. Data are from a German nationwide, cross-sectional study [26]. Only 11.5% of women supplemented, while 88.5% did not.

	Pregnant	Lactating	No Supplement	With Supplement
	*n* = 213	*n* = 127	*n* = 301	*n* = 39
EPA	0.52 ± 0.19%	0.72 ± 0.25%	0.58 + 0.24%	0.66 ± 0.20%
Range of EPA	0.12–1.12%	0.17–1.75%	0.12–1.75%	0.37–1.16%
DHA	6.10 ± 1.29%	4.86 ± 1.31%	5.45 + 1.36%	7.06 ± 1.17%
Range of DHA	3.46–10.29%	2.09–8.19%	2.09–10.29%	4.11–10.14%
Omega-3 Index	6.62 ± 1.39%	5.57 ± 1.39%	6.04 ± 1.39%	7.73 ± 1.28%
Range of Omega-3 Index	3.81–11.10%	2.49–9.24%	2.49–11.10%	4.61–11.08%
